# Optical breast atlas as a testbed for image reconstruction in optical mammography

**DOI:** 10.1038/s41597-021-01037-z

**Published:** 2021-09-30

**Authors:** Yidan Xing, Yubo Duan, Padmeya P. Indurkar, Anqi Qiu, Nanguang Chen

**Affiliations:** 1 Biomedical Engineering, National University of Singapore, Singapore, Singapore; 2 Hangzhou One-North Medical Technologies, Hangzhou, China; 3 Mechanical Engineering, National University of Singapore, Singapore, Singapore

**Keywords:** Imaging techniques, Biomedical engineering, Imaging and sensing

## Abstract

We present two optical breast atlases for optical mammography, aiming to advance the image reconstruction research by providing a common platform to test advanced image reconstruction algorithms. Each atlas consists of five individual breast models. The first atlas provides breast vasculature surface models, which are derived from human breast dynamic contrast-enhanced magnetic resonance imaging (DCE-MRI) data using image segmentation. A finite element-based method is used to deform the breast vasculature models from their natural shapes to generate the second atlas, compressed breast models. Breast compression is typically done in X-ray mammography but also necessary for some optical mammography systems. Technical validation is presented to demonstrate how the atlases can be used to study the image reconstruction algorithms. Optical measurements are generated numerically with compressed breast models and a predefined configuration of light sources and photodetectors. The simulated data is fed into three standard image reconstruction algorithms to reconstruct optical images of the vasculature, which can then be compared with the ground truth to evaluate their performance.

## Abstract (summary)

**Table:** 

Measurement(s)	breast vasculature surface model • compressed breast model
Technology Type(s)	image segmentation • finite element-based method
Factor Type(s)	patient
Sample Characteristic - Organism	Homo sapiens

Machine-accessible metadata file describing the reported data: 10.6084/m9.figshare.16477779

## Background & Summary

Optical mammography (OM) based on diffuse optical tomography (DOT) is an emerging medical imaging modality for non-invasive, functional imaging of human breast^1^. Its potential applications include breast cancer screening, optical biopsy, and treatment monitoring^2^. DOT provides tomographic maps of optical properties (the absorption and reduced scattering coefficients), which can be translated to physiological parameters such as blood volume and blood oxygenation. The image formation process of DOT is complicated and challenging. To make non-invasive optical measurements, the light sources and photodetectors are deployed to illuminate the body surface and collect diffusive reflectance and/or transmittance from the surface as well. An image reconstruction algorithm has to be employed to convert the surface measurements to tomographic images.

The past two decades have witnessed a continuous effort to improve the imaging performance of DOT in terms of spatial resolution, reconstruction accuracy, and robustness. Researchers have been addressing the existing problems with DOT from two aspects: optical instrumentation and image reconstruction^3^. Optical instruments generally fall into three categories: the continuous wave, frequency-domain, and time-domain methods. Each instrumentation method has its advantages and disadvantages in comparison with others. For example, a time-domain system generally performs better than a continuous-wave system in terms of image quality. However, a continuous wave system is usually less costly and less complex to build and therefore can be equipped with more measurement channels (i.e., source-detector pairs).

The major mathematical problem in DOT is to generate maps of optical properties from the measurements. The so-called image reconstruction is essentially an inverse problem^4^. For many other medical imaging techniques, it is straightforward to solve the inverse problem once the forward model is properly established. In the case of DOT, however, the inverse problem is generally ill-posed and therefore much more challenging due to the strong scattering of photons in human tissue.

There have been numerous image reconstruction algorithms reported previously^5^, such as linear methods (i.e., Moore-Penrose pseudoinverse^6^, Tikhonov regularization^7^, and compressive sensing^8^), and non-linear iterative methods (i.e., Gauss-Newton optimization^9,10^). Recently, there is a growing interest in revolutionizing conventional reconstruction algorithms with artificial intelligence. For example, a convolutional neural network (CNN) has been applied to the DOT image reconstruction, providing a data-driven approach towards optimal regularization^11^.

For researchers working on image reconstruction techniques, one of the challenges is the lack of a suitable platform for testing and quantitatively evaluating reconstruction algorithms. Experimental data is not widely available to the public. To make the situation even worse, the data is usually acquired with custom-designed instruments rather than commercial products following industrial standards. It is therefore questionable if an algorithm optimized for a specific instrument will also perform equally well for other systems. Not to mention various types of human errors during the clinical experiments. Computer simulation for data generation is a popular choice due to its simplicity and repeatability in comparison with the experimental approach. The tissue models involved in the simulation, however, have been over-simplified so far. The breast tissue is generally considered homogeneous except for a few small inhomogeneities (*e.g*., cuboids and spheres). In this work, we present much more realistic breast models that are derived from human breast DCE-MRI data. DCE-MRI is an *in vivo* imaging method providing information about the vasculature, which contains the major near-infrared (NIR) absorbing molecules (*i.e*., hemoglobins). Our breast atlases are essentially image databases that define the surfaces of blood vessels. In the future, we will “implant” abnormalities such as tumors in the breast atlas to investigate the capability of DOT in differentiating tumors from normal blood vessels, which is very important for improving diagnostic accuracy. The breast atlases can be used to generate a huge number of simulated data that better mimic the experimental data from human subjects with a wide range of experimental setups. To demonstrate the usefulness of the optical breast atlases, we further provide simulated measurement data obtained from compressed breast models as technical validation. Three standard image reconstruction algorithms are utilized to perform image reconstruction. The quality of reconstructed images is then assessed by comparing them with the ground truth.

## Methods

### MRI image processing procedures

The breast models are constructed from the previously published human breast DCE-MRI dataset which is publicly available on *the cancer imaging archive*^12,13^. The data is from 10 patients (numbered 01, 05, 06, 08, 10, 12, 13, 14, 15, and 16) with local breast cancer who undergo chemotherapy. The data available on the cancer imaging archive was acquired at two time points: prior to the start of treatment (visit 1, V1) and after the first cycle of treatment (visit 2, V2). We use the data at V1 for this study. The three-dimensional gradient echo-based TWIST (time-resolved angiography with stochastic trajectories) sequence was used to acquire the axial bilateral DCE-MRI images with fat saturation and full breast coverage^14^. The DCE-MRI acquisition parameters listed in the reference include^13,14^: 10° flip angle, 2.9/6.2 millisecond echo time/repetition time (TE/TR), a parallel imaging acceleration factor of two, 30 to 34 cm field of view (FOV), 320 × 320 in-plane matrix size, ~1.0 × 1.0 × 1.4 mm^3^ spatial resolution, ~10 minutes total acquisition time, 18 s and 20s temporal resolutions for 120 and 128 slices respectively. At the beginning of the 3^rd^ image acquisition, gadolinium-based contrast agent (ProHance) started to be injected.

**Fig. 1:**
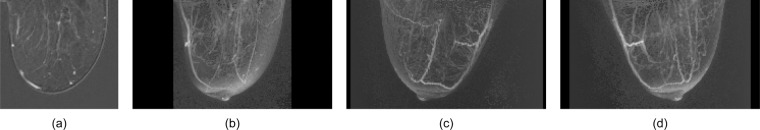
One example of the basic breast atlas: (**a**) a 2D slice; (**b**–**d**) maximum projections in three different angles.

To reduce the background influence of the MRI image, we processed the breast individually and cropped a region of interest covering one of the breasts from the original DCE-MRI images. The region of interest was used for all the subsequent processing for a single breast of each patient. To maximize the contrast of breast DCE-MRI data, we chose the DCE-MRI images within a certain time range during which the intensity changes due to the contrast agent reach the maximum. Each one of the selected images was subtracted by a baseline image, which was acquired before the injection of the contrast agent. The difference images were then averaged over the time range to reduce the background noise and enhance the contrast. The resultant images with improved quality were considered as the basic breast atlas, which described the volumetric distributions of the contrast agent concentration inside the breast tissue. A widely used image processing software called ImageJ^15^ in biomedical engineering is used to check the quality of the basic breast atlas in terms of vasculature visibility. By viewing the atlas of 10 patients in volume viewer of ImageJ^15^, we observed that left breast of patient 06, both breasts of patient 08, both breasts of patient 10, both breasts of patient 14, and left breast of patient 16 have relatively higher vasculature contrast and show more complex vasculature structures. From these 8 individual breasts, we first chose those without tumor (i.e., left breast of patient 06, right breast of patient 10, and left breast of patient 14). Besides that, we chose the one with the smallest tumor (left breast of patient 08) and the one with the largest tumor (right breast of patient 08). The 5 selected individual breasts will be referred to as 06L, 10R, 14L, 08L, and 08R respectively hereafter. The basic breast atlas can be found in our dataset “basic breast atlas” folder. As an example, the basic breast atlas of 10R is displayed in Fig. 1(a–d) in the views of a 2D slice in the z-axis, and maximum projections in three different angles that are rotated around the y-axis.

**Fig. 2:**
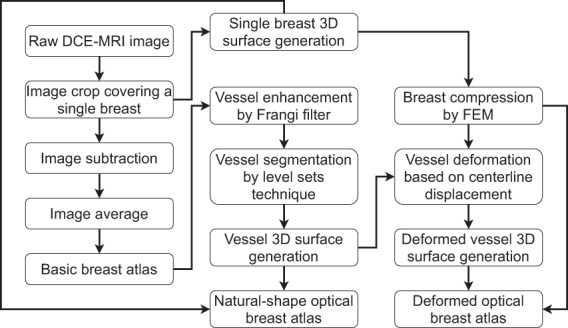
Diagram for the generation of optical breast atlases.

The contrast agent concentration is not directly linked to hemoglobin concentrations, as the contrast agent may leak from the vascular space to the extravascular extracellular space. Consequently, it is non-trivial to use the basic breast atlas directly in optical simulations. A complex mapping might be needed to assign optical properties based on local image intensity. In this work, we focus on generating atlases (i.e., natural-shape and deformed breast atlas) that are simpler to use. The framework for generating the atlases is illustrated in Fig. 2.

### Natural shape optical breast atlas

While the optical breast atlases are mainly used to define the vasculature (and therefore hemoglobin concentration) in breasts, it is also important to provide geometric information for the breast surfaces. The boundary geometry is necessary for the proper configuration of sources and detectors on the breast surface. It is also important for assigning optical properties for the background breast tissue as well as those of the matching medium (if any).

**Fig. 3:**
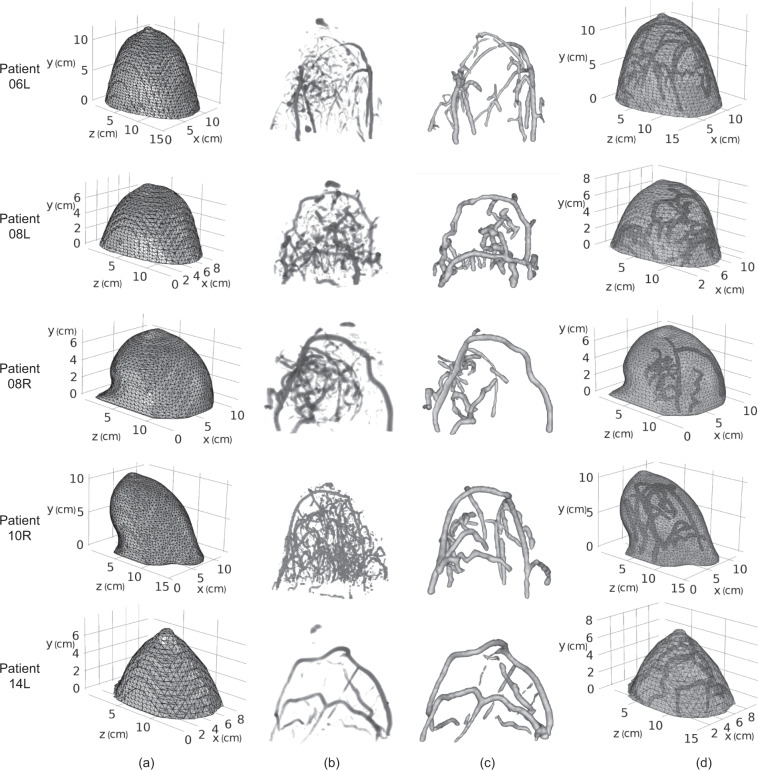
For patients 06L, 08L, 08R, 10R, and 14L: (**a**) breast 3D surfaces in triangulated meshes; (**b**) the basic breast atlas after vessel enhanced by Frangi filter; (**c**) Vessel 3D surfaces segmented by VMTK level sets segmentation; (**d**) the natural-shape optical breast atlas in triangulated meshes.

We used a selected set of MRI images to construct the breast 3D surfaces. They were chosen from the raw DCE-MRI image sequence that has been used for generating the basic atlas. ImageJ^15^ is used to find the breast iso-surface by setting an appropriate threshold to divide the breast and the background. The breast 3D surfaces are constructed in ImageJ^15^ by the marching cubes algorithm^16^. They are saved as triangulated mesh files in our dataset “natural-shape breast atlas” folder. In Fig. 3, column (a) shows the five individual breast 3D surfaces in triangulated meshes.

The first optical breast atlas was created by applying image segmentation techniques to the basic breast atlas. It includes five breast models with vessels constructed for patients 06L, 08L, 08R, 10R, and 14L. When using the optical breast atlas for optical simulation, different optical properties (i.e., absorption and reduced scattering coefficients) can be pre-defined for the vessel and non-vessel (background) tissue regions inside the breast models accordingly. To define the geometry of the vascular network, vessel 3D surfaces were generated using image segmentation techniques: Frangi vessel enhancement, and level sets segmentation.

Frangi filter^17^ is one of the most popular methods for human vasculature visualization. It works well in vessel enhancement and background suppression in the volumetric display. In this work, the Frangi filter was used as the pre-processing step for vessel segmentation. Frangi filter examines the second-order local structure of an image based on the eigenvalues of Hessian. The relationships between the magnitude of eigenvalues of Hessian can be interpreted into line-like, blob-like, and plate-like geometric structures. Besides, the norm of the Hessian can be associated with the background where the eigenvalues are small due to low contrast. The Frangi filter defines a metric to measure the vessel likeliness by distinguishing the line-like structure from the blob-like and plate-like structures and meanwhile suppressing the background. Using the Frangi filter, the vessel structures are enhanced in the basic breast atlas. In Fig. 3, column (b) shows the volumetric display of the basic breast atlas after vessel enhanced by Frangi filter for the five individual breasts, which can be found in our dataset “natural-shape breast atlas” folder.

Subsequently, we applied the level sets technique^18^ to the vessel-enhanced images for the vessel segmentation. The level sets technique involves an evolution of a curve ξ(t), which is represented by a zero-level set of a higher dimensional function. This curve can dynamically change its topology: split and merge, which makes the level sets technique especially useful in vessel segmentation^19^. An evolving surface *γ*(t) is represented as a level set function *γ*(t) = {**x|***φ*(**x**, t)}. The zero-level curve is given by $\xi ({\rm{t}})=\{{\bf{x}}| \varphi ({\bf{x}},{\rm{t}})=0\}$, for region bounded by the zero-level curve: $\{{\bf{x}}| \varphi ({\bf{x}},{\rm{t}}) < 0\}$, and for the region outside the zero-level curve: $\{{\bf{x}}| \varphi ({\bf{x}},{\rm{t}}) > 0\}$. The surface *γ*(t) propagates in time along its normal direction with a speed function driven by the surface geometry (such as curvature) and image attributes (such as gradient). The evolution equation of the level set function *φ*(**x**, t) is written in a general form:

1 $$\frac{\partial \varphi ({\bf{x}},{\rm{t}})}{\partial t}+F({\bf{x}},{\rm{t}})\left|\nabla \varphi ({\bf{x}},{\rm{t}})\right|=0,$$

where *F*(**x**, t) denotes the speed function in the normal direction of the surface *γ*(t). The speed function used in this work is determined by propagation, smoothing, and advection terms, denoted by *G*(**x**), *H*(**x**), and ∇*P*(**x**) respectively^20,21^.

2 $$F({\bf{x}},{\rm{t}})={w}_{1}G({\bf{x}})-{w}_{2}H({\bf{x}})-{w}_{3}\nabla P({\bf{x}})\cdot \frac{\nabla \varphi }{\left|\nabla \varphi \right|}.$$

The propagation term *G*(**x**) represents the surface inflation speed and is determined by image edge information. The smoothing term *H*(**x**) is determined by the mean curvature of the surface. The advection term ∇*P*(**x**) represents the attraction potential to the ridges of image gradient magnitude. The level sets technique has been intensively studied and well implemented by researchers. The vascular modeling toolkit (VMTK)^22^ (http://www.vmtk.org) is software available online developed specifically for image-based 3D modeling of vessels. In this work, we used the software VMTK to implement vessel segmentation. To reduce the manual operations, we used the threshold as the initialization type. Pixels with intensities within two user-given thresholds were selected as the initial zero-level curve. Parameters *w*_1_, *w*_2_, *w*_3_ that control the effect of propagation, curvature, and advection terms to the level sets evolution were given specifically for each individual breast. The stopping criterion was subject to the number of iterations given by users based on experience. The vessels were segmented from the vessel-enhanced image by the VMTK level sets segmentation, followed by manual erasing of noisy volume in ImageJ. Same as the breast surface, the resultant vessel 3D surfaces are shown in column (c) of Fig. 3 for the five individual breasts and saved as triangulated mesh files in our dataset “natural-shape breast atlas” folder.

Finally, the breast and vessel 3D surfaces were combined into a composite model with the Boolean difference to avoid geometry overlapping. The resultant composite model is a natural shape breast model, in which the non-vessel and vessel tissues can be assigned with different sets of optical properties for optical mammography simulation. Column (d) of Fig. 2 shows the natural shape breast models for patients 06L, 08L, 08R, 10R, and 14L, which can be found in our dataset “natural-shape breast atlas” folder. The sizes (in cm) of their bounding boxes in the z, y, and x-axis are listed in Table 1.

**Table 1:** For patients 06L, 08L, 08R, 10R, and 14L: sizes of bounding boxes of optical breast atlases in z, y, and x-axis (unit: cm).

	Natural shape optical breast atlas	Deformed optical breast atlas	Optical simulation configuration
Patients 06L,	(14.58, 12.35, 13.10)	(21.27, 16.38, 4.74)	(27.27, 22.38, 5.00)
Patients 08L,	(13.79, 8.14, 9.78)	(16.91, 9.87, 4.87)	(22.91, 15.87, 5.00)
Patients 08R	(13.07, 7.66, 10.83)	(18.42, 10.39, 4.35)	(24.42, 16.39, 4.50)
Patients 10R	(15.29, 10.85, 11.18)	(20.74, 13.98, 4.70)	(26.74, 19.98, 5.00)
Patients 14L	(14.30, 8.15, 9.61)	(17.53, 9.50, 4.90)	(23.53, 15.50, 5.00)

### Deformed optical breast atlas by compression

In breast mammography, the source-detector separation is a very important parameter. NIR light is known to be able to penetrate the breast tissue for a few centimeters. Therefore, source-detector pairs with a large distance usually are not useful as the detected photons could be overwhelmed by noises. This places a practical constraint on the configuration of light sources and photon detectors, especially for imaging a human breast in its natural shape. In some optical mammography methods, the breast under investigation is slightly compressed and flattened so that the distances between the sources and detectors are significantly reduced in the transmittance measurements. Besides the advantage in signal integrity, the planar surfaces of a compressed breast lead to simplified model geometry and therefore reduced computational cost. Sometimes even analytic solutions to the diffusion equation can be used as a good approximation.

To facilitate algorithm development for optical mammography based on the breast compression strategy, we further created the deformed optical breast models to increase the variety of the optical breast atlases. In the deformed optical breast atlas, each breast model is a compressed version of its counterpart in the natural-shape optical breast atlas. Its thickness between two compression plates was gradually reduced to less than 5 cm by the use of a finite element method (FEM), which is known for strain deformation analysis.

### Breast compression by FEM

**Fig. 4:**
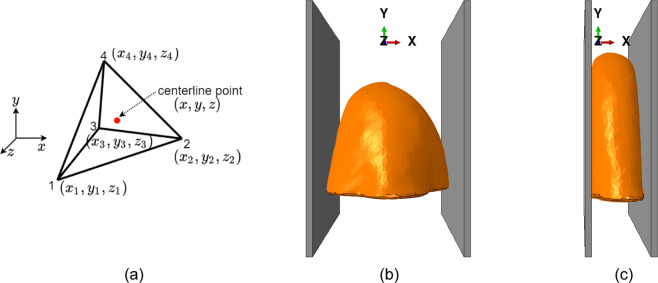
(**a**) linear tetrahedral finite element (C3D4) used for discretization of the breast body; The FEM simulation setups (**b**) before the breast compression, (**c**) after the breast compression.

We used a well-known finite element analysis software, ABAQUS/Standard, for breast compression. The breast surface geometry was filled with a volume of homogeneous material and converted to a 3D deformable solid body. In FEM, the object domain, denoted by Ω, was divided into non-overlapping subdomains, the so-called finite elements. When using *τ*_*i*_ to represent the *i*^th^ element, the object domain was expressed as $\Omega ={\cup }_{i=1}^{L}{\tau }_{i}$, where *L* was the total number of elements. The 3D breast geometry was meshed with ~90000 linear tetrahedral finite elements (C3D4) for conducting large strain deformation analysis. The vertices of the element were called nodes. As shown in Fig. 4(a), the C3D4 has four nodes. The linear tetrahedron is one of the simplest finite elements and it is very flexible in discretizing geometry shape. In addition, using the linear tetrahedron can reduce the large computational load of the large deformation. Hyperelastic neo-Hookean constitutive material model from the ABAQUS® library was adopted for the breasts. The neo-Hookean strain energy potential was determined, given Young’s modulus *E*^breast^ = 0.5 KPa and the Poisson’s ratio *v*^breast^ = 0.49, according to the mechanical properties of the breast in reference^23,24^. To simulate the breast compression scenario, two 3D plate sections were modeled along the lateral ends of the breast geometry. This plate was assigned with elastic and plastic properties resembling steel (i.e., Young’s modulus *E*^plate^ = 210 GPa and Poisson’s ratio *v*^plate^ = 0.33). The plate sections were one element thick and meshed with linear brick elements (C3D8).

In the FEM simulation, we aimed to compress the breast body to less than 5 cm in thickness. Friction-based surface contact formulation between the plates and the breast was adopted, with a slip tolerance of about 0.01. One of the plates was made stationary by arresting its translation and rotational degrees of freedom, while a velocity of 2500 mm *s*^−1^ was applied to the other plate which compresses the breast body. The degrees of freedom of the nodes on the base of the breast were also arrested to avoid rigid rotations and translation while allowing a contraction of the breast. The simulation set-ups, together with one individual breast before and after the compression, are shown in Fig. 4(b,c) respectively. The reaction force experienced by the fixed plate was found to be ~100 N for the breast body, which corroborates well with the clinical compression force^25^. The resultant compressed breast 3D surfaces for patients 06L, 08L, 08R, 10R, and 14L  can be found in our dataset “deformed breast atlas” folder.

### Vessel deformation

**Fig. 5:**
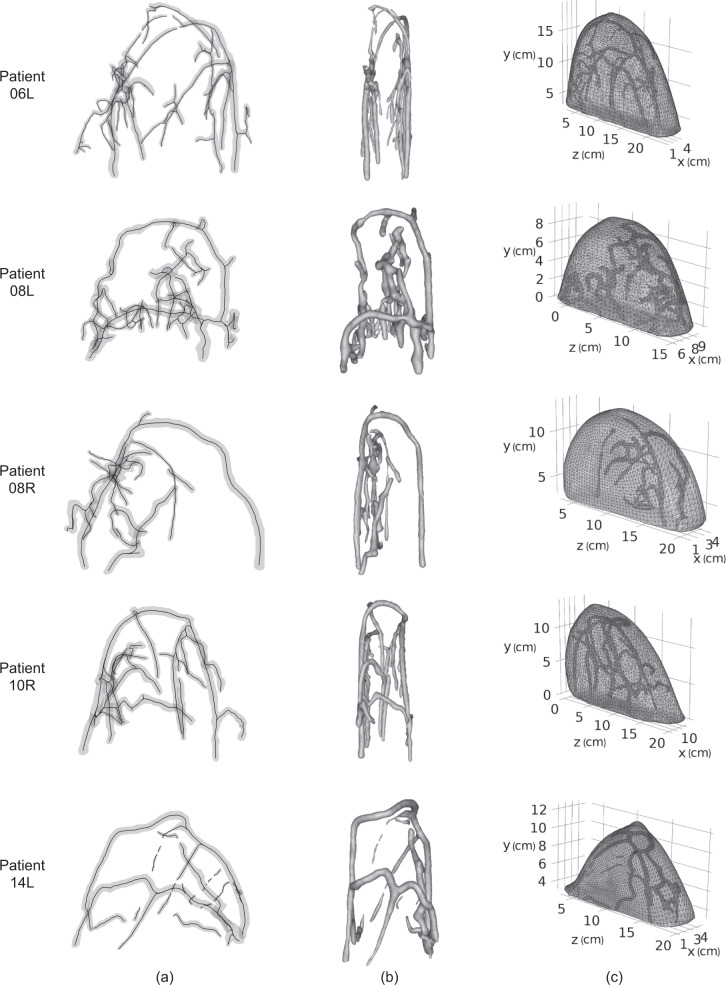
For patients 06L, 08L, 08R, 10R, and 14L: (**a**) centerlines of vessel surfaces extracted in VMTK; (**b**) the deformed vessel surfaces; (**c**) the deformed optical breast atlas.

To deform the vessel structures according to the breast compression process while maintaining their tubular shapes, we considered the deformation of vessel centerlines, rather than that of vessel surfaces. The centerline plays an important role in describing the vascular structures. It is usually defined as the line drawn from two extremal points of a vessel branch which locally maximizes the distance from the vessel boundary. Therefore, we proceeded with the vessel centerline extraction based on the vessel surface constructed. We used the software VMTK to extract the centerlines of the vessels from the natural-shape optical breast atlas. In VMTK, the centerline calculation is formulated on the Voronoi diagram domain, which is the distance transform of the vessel surface. On the Voronoi diagram, Voronoi spheres are defined as the maximal inscribed spheres with respect to the point set sampling the vessel surface. The centerline is calculated by minimizing the integral of the inverse of the radius of Voronoi spheres along a path between two given points. This minimization is achieved by computing the shortest path on the Voronoi diagram between the two given points in the metric of the inverse of the radius of Voronoi spheres. Based on the vessel surfaces shown in column (c) of Fig. 3, their centerlines were extracted in VMTK and shown in column (a) of Fig. 5 for patients 06L, 08L, 08R, 10R, and 14L.

Since we used the linear tetrahedral finite element (C3D4) for the discretization of the breast body in the breast compression simulation by FEM, the linear interpolation basis functions were used to calculate the deformation of centerlines of the vessels. The breast compression simulation was based on the deformation at every node. The deformation of points sampling the centerlines was calculated by linear interpolations of the nodes’ deformation. As shown in Fig. 4(a), the four nodes of a tetrahedral element have local and global coordinates in the cartesian system. Each node has a linear basis function, whose value varies from 0 to 1. The basis functions are expressed by

3 $${{\rm{\psi }}}_{j}({\rm{x,\; y,\; z}})={a}_{j}+{b}_{j}x+{c}_{j}y+{d}_{j}z,\quad j=1,2,3,4.$$

The coefficients ${a}_{j},{b}_{j},{c}_{j},{d}_{j},\;j=1,2,3,4$ can be obtained from the global coordinates of the four nodes (x_*j*_, y_*j*_, z_*j*_) using the property that the basis function equals 1 at its node and equals 0 at all other nodes (i.e., ${{\rm{\psi }}}_{j}({{\rm{x}}}_{j}{{\rm{,\; y}}}_{j}{{\rm{,\; z}}}_{j})=1$ and ${{\rm{\psi }}}_{j}({x}_{i},{y}_{i},{z}_{i})=0(j\ne i)$). The specific expressions for coefficients ${a}_{j},{b}_{j},{c}_{j},{d}_{j}$ can be found in reference^26^. Once the basis functions known, the deformation of any point located inside an element can be approximated by a piecewise linear and continuous function,

4 $${u}^{h}(r)=\mathop{\sum }\limits_{j=1}^{4}{u}_{j}{{\rm{\psi }}}_{j}(r),$$

where ${u}^{h}(r)=(\Delta x,\Delta y,\Delta z)$ is the deformation of the point to be interpolated in *x*, *y*, *z* directions. ${u}_{j}=(\Delta {x}_{j},\Delta {y}_{j},\Delta {z}_{j}),j=1,2,3,4$ are the deformation of the element’s four nodes during breast compression.

The vessel structures were deformed according to the deformation of centerlines. It was assumed that the deformed vessel surface was still composed of all the triangulated mesh nodes on the natural vessel surface but with a translation. For each mesh node, the translation was determined by the deformation of the point sampling the centerlines that was closest to the mesh node. Specifically, if *P*_*s*_ was a mesh node on the natural vessel surface, and *P*_*c*_ was the closest sampling point on centerlines to *P*_*s*_, then *P*_*s*_ was translated by the deformation of *P*_*c*_ and became a mesh node for the deformed vessel surface. The deformation of sampling points on centerlines was calculated by Eq. (4). Similarly, every mesh node on the natural vessel surface was translated to constitute the deformed vessel surface. It is worthy to note that the deformed vessel surface generated might contain errors in a triangulated mesh that make the part unusable for a FEM problem. We applied automatic repair of Autodesk Netfabb to the deformed vessel surface. The repair action includes deleting self-intersecting faces, stitching triangles, closing trivial holes, etc. The natural vessel surface in column (c) of Fig. 3 is deformed according to the deformation of centerlines in column (a) of Fig. 5. The resultant deformed vessel 3D surfaces are shown in column (b) of Fig. 5 for patients 06L, 08L, 08R, 10R, and 14L and saved as triangulated mesh files in our dataset “deformed breast atlas” folder.

The deformed vessel surfaces were embedded into the compressed breast surfaces and became the deformed breast models. Column (c) of Fig. 5 shows the deformed breast models for patients 06L, 08L, 08R, 10R, and 14L, which can be found in our dataset “deformed breast atlas” folder. The sizes of their bounding boxes in the z, y, and x-axis are listed in Table 1. The deformed optical breast atlas consists of the five deformed breast models, each of which is a composite geometric model defining both the surface of the compressed breast body and surfaces of the enclosed blood vessels.

## Data Records

This optical breast atlas dataset is publicly available on *figshare*^27^ which contains sixteen zip files. The name and description of each file are as follows.

- “basic breast atlas” folder
  For each of the five individual breasts:
  “<patient no.>_<basic>.tif”: the basic optical breast atlas.
- “natural-shape breast atlas” folder
  For each of the five individual breasts:
  “<patient no.>_<breastSurface>.stl”: the natural-shape breast 3D surfaces.
  “<patient no.>_<f>.tif”: the basic breast atlas after Frangi filter.
  “<patient no.>_<ls>.tif”: the basic breast atlas after Frangi filter and level sets segmentation.
  “<patient no.>_<vesselSurface>.stl”: the natural-shape vessel 3D surfaces.
  “<patient no.>_<natural>.x_t”: the natural-shape optical breast atlas.
- “deformed breast atlas” folder
  For each of the five individual breasts:
  “<patient no.>_<defBreastSuface>.stl”: the deformed breast 3D surfaces.
  “<patient no.>_<defVesselSurface>.stl”: the deformed vessel 3D surfaces.
  “<patient no.>_<deform>.x_t/.SLDPRT”: the deformed optical breast atlas.
- “simulation_configuration” folder
  For each of the five individual breasts:
  “<patient no.>_<cuboid>.sldprt”: the deformed breast models in the cuboid containers.
  “<patient no.>_<sim>.mph”: the COMSOL Multiphysics optical simulations.
- Five “simulation_measurement_<patient no.>” folders
  Each folder contains the simulated surface measurements with vessel inhomogeneity
  for each of the five individual breasts:
  “t_<source no.>.mat”: transmittance images with a resolution of 0.025 cm × 0.025 cm for each source location.
  “r_<source no.>.mat”: reflectance images with a resolution of 0.025 cm × 0.025 cm for each source location.
  For example, in “simulation_measurement_08L” folder, “r_1.mat” and “t_1.mat” refer to the reflectance and transmittance images corresponding to source no. 1, for patient 08L with vessel inhomogeneity.
- Five “simulation_measurement_<patient no.>_background” folders
  Each folder contains the simulated surface measurements without vessel
  inhomogeneity for each of the five individual breasts.
- “simulation_mua_reconstructed” folder
  For each of the five individual breasts:
  “<patient no.>_mua_<method>.mat”: the maps of absorption coefficients reconstructed by the Tikhonov, compressive sensing with L1-norm, and compressive sensing with total variation methods. For example, “08L_mua_tik.mat” refers to the map of absorption coefficients reconstructed by the Tikhonov method for patient 08L.
- “simulation_mua_ground truth” folder

For each of the five individual breasts:

“<patient no.>_mua_gt.mat”: the ground truths of absorption coefficients

## Technical Validation

The deformed optical breast atlas was employed to perform computer simulations and generate simulated optical measurements, which would be later used as inputs to image reconstruction algorithms.

### Diffusion equation

It is generally accepted that the propagation of electromagnetic waves in scattering medium can be described by the radiative transfer equation (RTE)^28,29^. However, the RTE is too complicated to obtain analytical solutions that can be generalized for solving real problems, such as a non-homogenous medium with complex geometry. In terms of numerical solutions to the RTE, the computational burden is very heavy. Therefore, diffusion approximation to the RTE is commonly used for an optically thick medium, in which multiple scattering events occur. With the diffusion approximation, the RTE is simplified to the diffusion equation (DE). The time-dependent DE is expressed as,

5 $$\frac{1}{c}\frac{\partial \phi ({\rm{r,\; t}})}{\partial t}-D{\nabla }^{2}\phi ({\rm{r,\; t}})+{\mu }_{a}\phi ({\rm{r,\; t}})=S({\rm{r,\; t}})$$

where *ϕ*(r, t) is the fluence rate, *c* is the speed of light inside the medium, *μ*_*a*_ is the absorption coefficient, and *S*(r, t) is the source term. *D* is the diffusion coefficient. It is given by $D={[3\left({\mu }_{s}{\prime} +{\mu }_{a}\right)]}^{-1}$, where ${\mu }_{s}{\prime}$ is the reduced scattering coefficient.

### Boundary conditions

Since the sources and detectors are placed on the surface of the object under investigation, the solution to the DE is subject to boundary conditions at the object surfaces. The typical boundary condition, which is called partial-current boundary condition, is given by^30^

6 $$\phi ({\rm{r,\; t}})=2AD\widehat{n}\cdot \nabla \phi ({\rm{r,\; t}}),$$

where *r* is on the object boundary surfaces, $A=(1+R)/(1-R)$ with *R* being the effective internal reflection coefficient and representing the fraction of the incident light upon the boundary surface that is reflected back into the object, $\widehat{n}$ is the unit vector normal to the boundary surface inwardly pointed. ∇*ϕ*(r, t) is the normal derivative of the fluence rate.

### FEM-based DE solver

As one of the well-known numerical methods to solve the DE, FEM has the advantage of being computational fast and applicational flexible compared to the Monte Carlo and analytical methods. In this work, we adopted a FEM-based photon propagation model to solve the DE for the geometry derived from the deformed optical breast atlas.

**Fig. 6:**
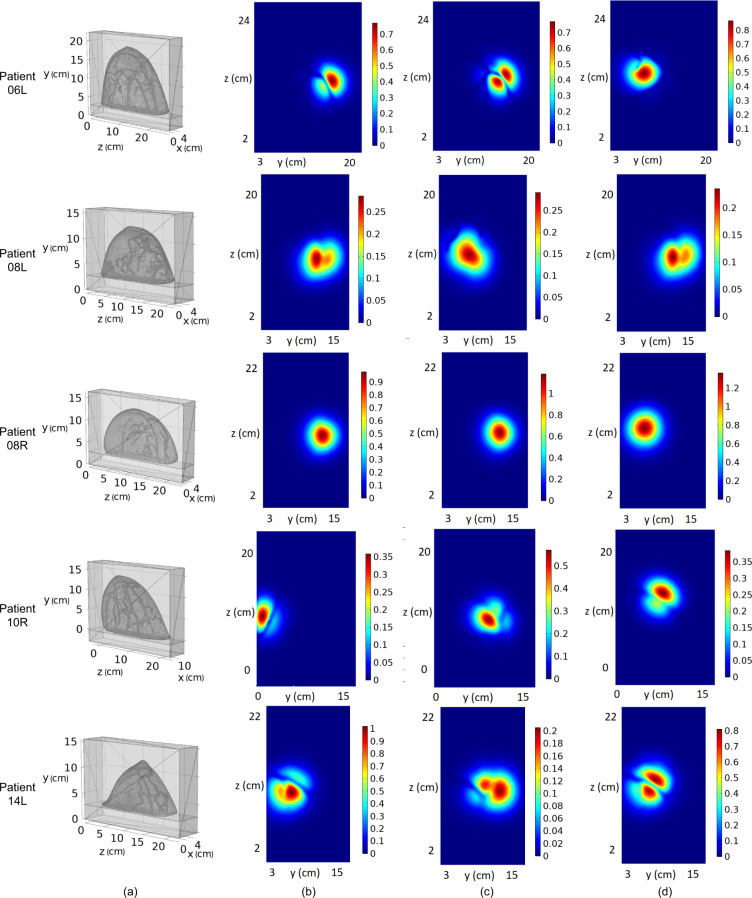
For patients 06L, 08L, 08R, 10R, and 14L: (**a**) simulation configuration: the cuboids containing deformed optical breast atlas; (**b**–**d**) examples of simulation results: three ransmittance images.

We used a commercial PDE solver, called COMSOL Multiphysics, to simulate photon propagation in a cuboid that contained a deformed optical breast model. Each cuboid included a slab of tissue used to mimic the chest wall behind the breast. A matching medium was used to fill the remaining space of the cuboid. The cuboid was created, whose size was determined according to the bounding box of the deformed breast model. We extended the margins of the bounding box of the deformed breast model by 3 cm along the lateral (y and z) directions. The cuboid was split into two compartments by a plane that was parallel to the x-z plane. The compartment with a width of 2.9 cm was used to mimic the chest wall and the other compartment contained the compressed breast model as well as the matching medium with similar optical properties as the breast tissue. In Fig. 6, column (a) presents the resultant cuboids for patients 06L, 08L, 08R, 10R, and 14L, and the sizes in the z, y, and x-axis are listed in Table 1. The simulation configuration, including the cuboid model and COMSOL application, can be found in our dataset “simulation_configuration” folder.

In the current work, we dealt with the stationary problem, i.e., $\partial \phi /\partial t=0$ in the time-dependent DE (Eq. (5)). The stationary DE can be solved using one of the classical PDEs, called the Helmholtz Equation, in COMSOL. First, the cuboid with the deformed breast model was imported into COMSOL and meshed with tetrahedral finite elements. In the cuboid, denoted by Ω, four domains were defined: breast, vessels, matching medium, and chest wall. Each domain was assigned with a particular set of optical properties (absorption and reduced scattering coefficients). We conducted the optical simulation at 900 nm and the optical properties used are listed in Table 2^31,32^. It is widely recognized that the female breast consists of vessels, adipose, glandular, and other tissues. Since the optical parameters of adipose, glandular and other tissues are relatively similar and the vessels deviate significantly from them^33–35^, we define the adipose, glandular, and other tissues as the homogenous surroundings (i.e, breast) and the vessels as the only heterogeneity (i.e., vessel). The partial-current boundary condition in Eq. (6), which is more generally called the Robin boundary condition, was used. Currently, we did not include the refractive index mismatch and assumed the refractive index within the cuboid (Ω) was the same as that of the surrounding medium^36^. In this case, there was no Fresnel reflection at the boundary surface, that was, the effective internal reflection coefficient *R* = 0 and the Robin boundary condition became:

7 $$\phi ({\rm{r,\; t}})=2D\widehat{n}\cdot \nabla \phi ({\rm{r,\; t}}).$$

**Table 2:** Absorption and reduced scattering coefficients for each domain of the cuboid.

Domain	Absorption coefficient *μ*_*a*_ cm^−1^	Reduced scattering coefficient ${\mu }_{s}^{{\prime} }$ cm^−1^
Breast	0.03	6
Vessel	4	20
Matching medium	0.06	8
Chest wall	0.1	10

The illumination light was considered as a Gaussian beam incident on the breast top surface. It was only after the beam entering the breast tissue and being scattered multiple times that the source could be treated as diffusive. Usually, an effective point-like diffusive source was used to model the illumination light. In this work, however, we chose to incorporate the source contribution directly into the Robin boundary condition:

8 $$\phi ({\rm{r,\; t}})=2D\widehat{n}\cdot \nabla \phi ({\rm{r,\; t}})+g({{\rm{r}}}_{0}{\rm{,\; t}}),$$

where *g*(r_0_, t) represented the influx of photons across the breast surface due to illumination centered at r_0_ on the boundary surface. The width of the Gaussian distributed continuous-wave (CW) beam was *σ*.

**Fig. 7:**
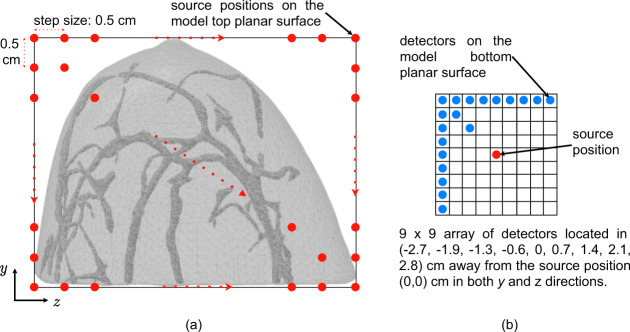
Souce-detector diagram: (**a**) source positions; (**b**) 9 × 9 array of detectors sampling the transmittance image.

As illustrated in Fig. 7(a), the illumination point r_0_ was raster scanned within the bounding box region of the breast on the model top planar surface at a step size of 0.5 cm in both *y* and *z* directions. With the domain-defined geometry, tetrahedral mesh, optical properties, and source integrated Robin boundary condition given above, it was straightforward to solve the DE for the fluence rate *ϕ*. The measurement signals collected by the detectors on the bottom planar surface were the flux values Γ, which were calculated from the fluence rate *ϕ* by the following equation,

9 $$\Gamma ({\rm{r,\; t}})=-D\nabla \phi ({\rm{r,\; t}}).$$

The flux can be well approximated by Eq. (9) for biological tissues when the CW source illumination was used. The simulated transmittance and reflectance signals could be then used for the 3D image reconstruction of optical properties inside the breast model. For each breast model, hundreds of transmittance and reflectance images were simulated for all the source positions, which can be found in our dataset “simulation_measurement_<patient no.>” folder. As examples, columns (b–d) of Fig. 6 show three transmittance images when the point source was located around the center of the scanning region for patients 06L, 08L, 08R, 10R, and 14L.

### Example reconstructed images

The transmittance images simulated in COMSOL were used for the 3D image reconstruction of optical properties. Since the ground truth of 3D optical properties inside the breast model was known, the reconstructed images could help quantitative assessment of the performance of different reconstruction algorithms. Therefore, the proposed optical breast atlas provides an efficient platform for a fair comparison between different reconstruction algorithms, which is the main motivation for this work. Generally, the reconstruction of the inhomogeneous breast model with complex vessel structures is a nonlinear inverse problem. However, a linearized approximation based on the perturbation theory has been widely used to estimate the small changes in optical properties from measured signal perturbations. The linear reconstruction algorithms based on the Jacobian matrix are the most basic yet important, and hence were employed in this work.

Based on the perturbation theory, the forward model (DE) can be linearized by the first Rytov approximation and expressed in the matrix form:

10 $$ln\left(\frac{\Phi }{{\rm{}}{\Phi }_{0}}\right)=\left[\begin{array}{cc}{{\rm{J}}}_{{\mu }_{a}} & {{\rm{J}}}_{D}\end{array}\right]\left[\begin{array}{c}\Delta {\mu }_{a}\\ \Delta D\end{array}\right],$$

where Φ_0_ and Φ are the measurement vectors for all source-detector pairs with background optical properties (*μ*_*a*0_, *D*_0_) and with spatial perturbations (*μ*_*a*0_ + Δ*μ*_*a*_, *D*_0_ + Δ*D*) respectively. Vectors Δ*μ*_*a*_ and Δ*D* are changes in absorption and diffusion coefficients at every voxel, respectively. J_*μa*_ and J_*D*_ are Jacobian matrices given by^37^

11 $${{\rm{J}}}_{{\mu }_{a}}(i,j)=-\frac{{{\rm{G}}}_{0}({{\rm{r}}}_{s},{{\rm{r}}}_{j}){{\rm{G}}}_{0}({{\rm{r}}}_{j},{{\rm{r}}}_{d})}{{{\rm{G}}}_{0}({{\rm{r}}}_{s},{{\rm{r}}}_{d})}dv,$$

12 $${{\rm{J}}}_{D}(i,j)=\frac{\nabla {{\rm{G}}}_{0}({{\rm{r}}}_{s},{{\rm{r}}}_{j})\cdot \nabla {{\rm{G}}}_{0}({{\rm{r}}}_{j},{{\rm{r}}}_{d})}{{{\rm{G}}}_{0}({{\rm{r}}}_{s},{{\rm{r}}}_{d})}dv,$$

where *i* is the index of a source-detector pair with source and detector indexes *s* and *d* respectively. *j* is the index of a voxel. *G*_0_(r_*s*_, r_*j*_) is the Green’s function with the background optical properties from the position of source *s* to the position of voxel *j*. *G*_0_(r_*j*_, r_*d*_) is the Green’s function from the position of voxel *j* to the position of detector *d*. *G*_0_(r_*s*_, r_*d*_) is the Green’s function from the position of source *s* to the position of detector *d*. *dv* is the volume of each voxel. Since the absorption and scattering coefficients cannot be determined simultaneously from a single-wavelength CW measurement^38,39^, only the absorption perturbations with depth compensation^40^ were considered in this current work.

Three linear reconstruction algorithms were employed for the reconstruction of absorption coefficients^2^. They were the Tikhonov regularization method, compressive sensing (CS) method with L1-norm, and CS method with total variation (TV). Considering the inevitable measurement noises in real experiments, 3% white Gaussian noises were added to the simulation measurements before the image reconstruction. The background absorption and reduced scattering coefficients were set to the same value for the breast domain. Considering the computational efficiency of image reconstruction, a 9 × 9 array of detectors was used to sample the transmittance image. The 9 × 9 array of detectors were spaced in a square that was centered to align with the source position, as illustrated in Fig. 7(b). The image reconstruction resolution was 0.4 × 0.4 × 0.4 cm^3^.

Tikhonov regularization method^7^ is based on the inversion of the Jacobian matrix. It addresses the Jacobian matrix’s ill-posed condition by adding an identity matrix with a regularization parameter *v*:

13 $$\Delta x={\left({{\rm{J}}}^{{\rm{T}}}\cdot {\rm{J}}+\nu {\rm{I}}\right)}^{-1}\cdot {{\rm{J}}}^{{\rm{T}}}\cdot \Delta {\rm{y}},$$

where $\Delta x=\Delta {\mu }_{a}$ is the perturbation of absorption coefficients for every voxel to be reconstructed, Δ*y* is the measurements for every source-detector pair Δ*y* = In(Φ/Φ_0_). *v* is chosen as $\nu =\widetilde{\nu }{{\rm{J}}}_{\max }$, J_max_ is the maximum diagonal element of the matrix J^T^·J, and $\widetilde{\nu }$ is a parameter relates to the reconstruction accuracy. $\widetilde{\nu }=0.01$ in this work.

CS is originally proposed for signal processing. It recovers signals from linear combinations of fewer signal samples than that required by the Shannon-Nyquist theorem^41^. The CS in optical image reconstruction is based on the prior information that the reconstructed optical coefficients vector is sparse in a particular transform domain^42^. This sparsity holds for the linear reconstruction methods since the linearized forward model assumes small optical perturbations in the model compared to the background state. CS with L1-norm solves the following minimization problem:

14 $$\Delta x=\arg \,{\min }_{\Delta x\in R}\left({\left\Vert {\rm{J}}\Delta x-\Delta {\rm{y}}\right\Vert }^{2}+\nu {\left\Vert \Delta x\right\Vert }_{1}\right),$$

where ${\left\Vert \Delta x\right\Vert }_{1}$ is the L1-norm of Δ*x*: ${\left\Vert \Delta x\right\Vert }_{1}={\sum }_{i}\left|\Delta {x}_{i}\right|$, *v* is the regularization parameter. As the name suggests, CS with TV changes the regularization term in the above equation to the TV of Δ*x*: ${\left\Vert \Delta x\right\Vert }_{{\rm{TV}}}={\sum }_{i}\left|\nabla (\Delta {x}_{i})\right|$. In this work, we assumed Δ*x* was a sparse vector and no sparsifying transform was used to further reinforce the sparsity since we deemed the vessel domain was spatially sparse compared to the homogeneous background. To solve the minimization problem, the TwIST algorithm^8^ was used, in which the solution was initialized as the result of the Tikhonov method.

**Fig. 8:**
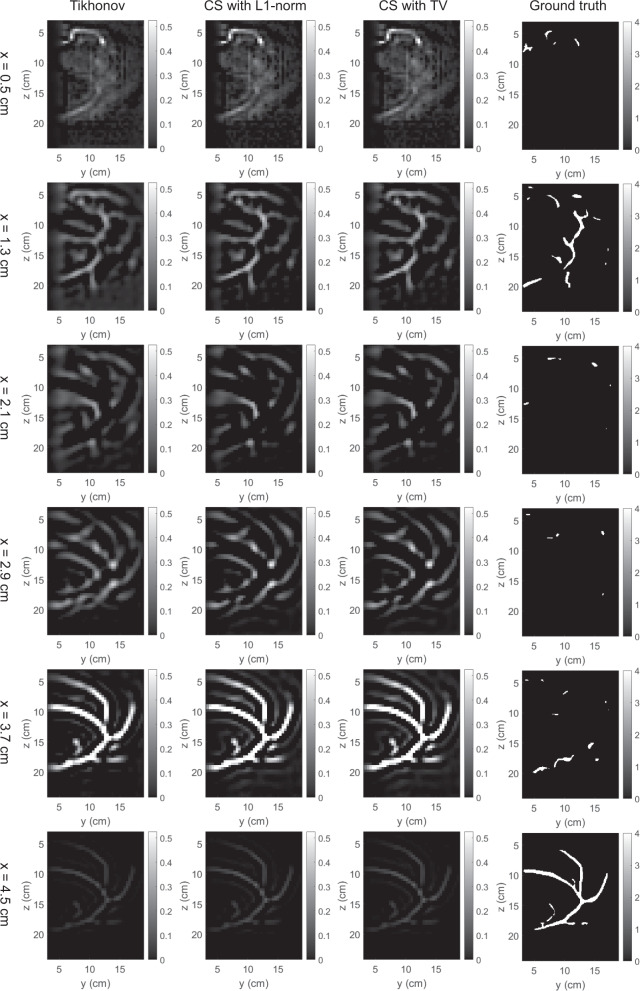
Absorption coefficients reconstructed for patient 06L.

**Fig. 9:**
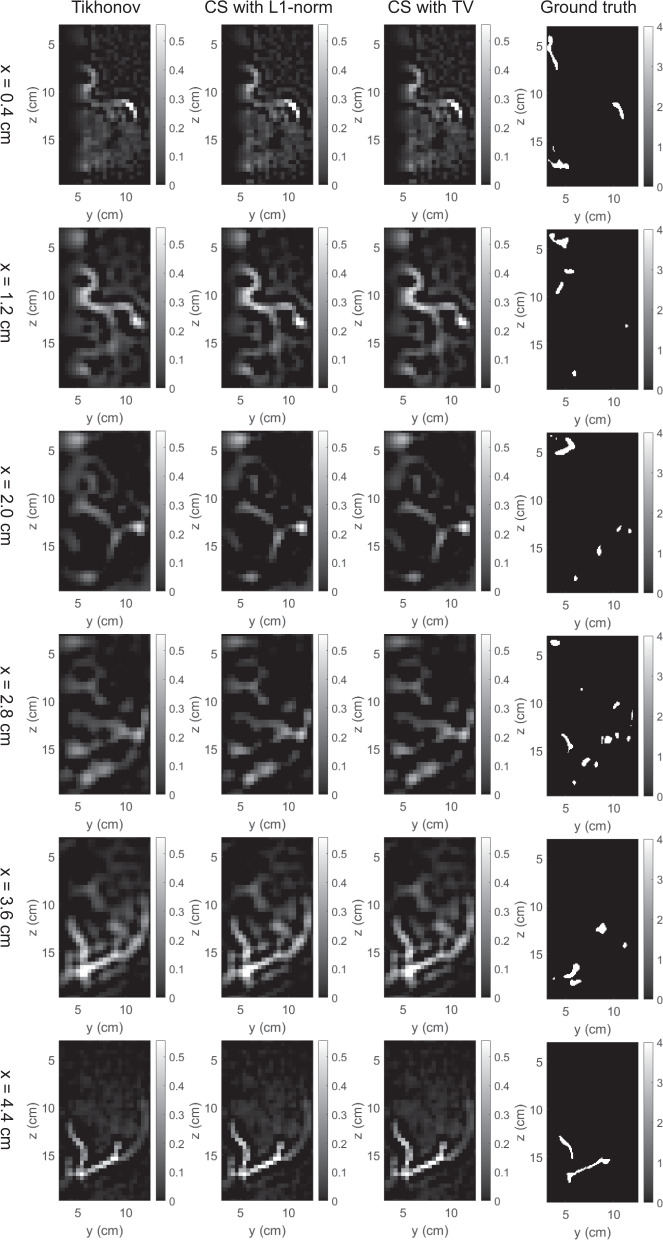
Absorption coefficients reconstructed for patient 08L.

**Fig. 10:**
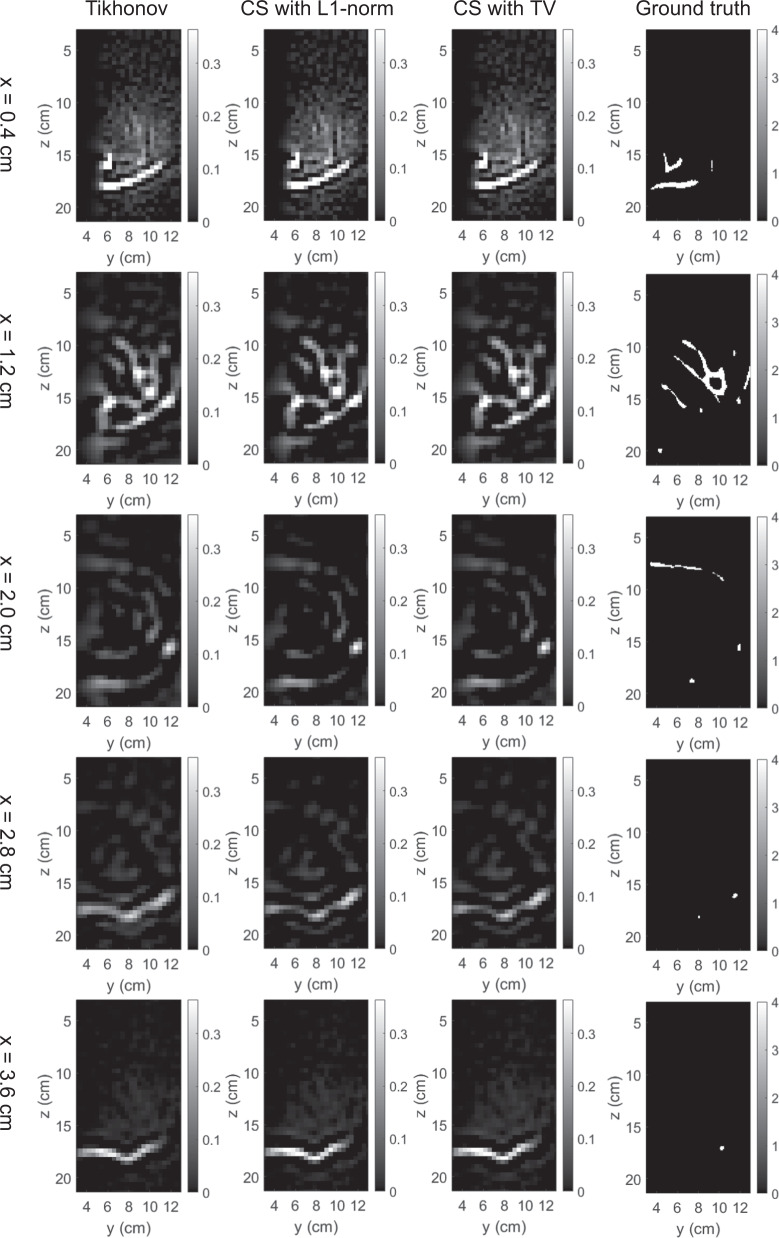
Absorption coefficients reconstructed for patient 08R.

**Fig. 11:**
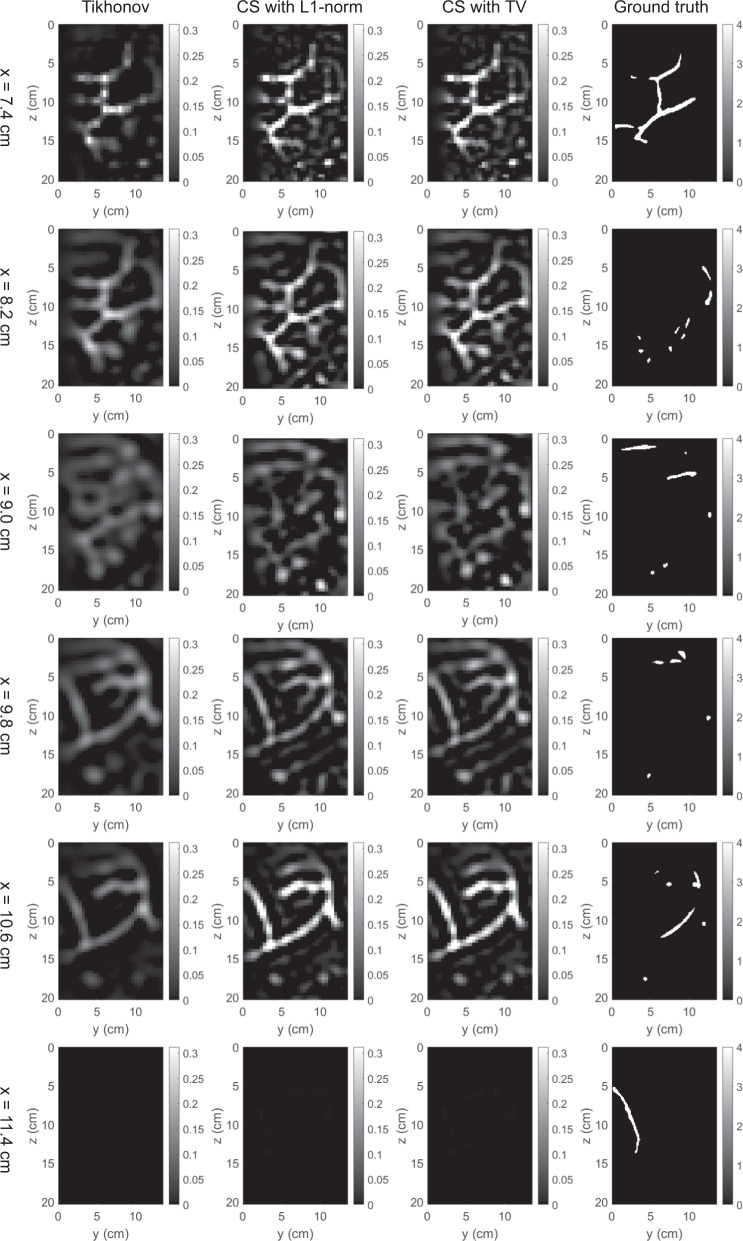
Absorption coefficients reconstructed for patient 10R.

**Fig. 12:**
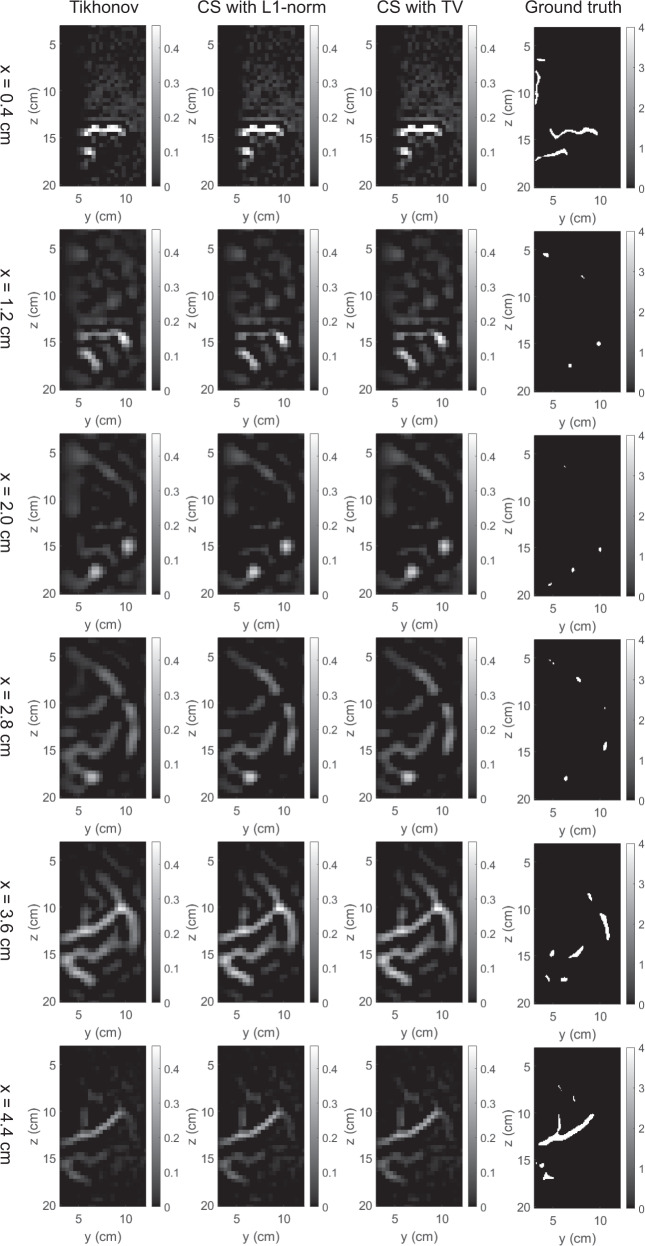
Absorption coefficients reconstructed for patient 14L.

The 3D map of absorption coefficients corresponding to the bounding box of the breast was reconstructed by the three methods. The reconstruction results were saved in our dataset “simulation_mua_reconstructed” folder. The ground truths of absorption coefficients according to the simulation configuration were exported from COMSOL and saved in our dataset “simulation_mua_ground truth” folder. Figures 8–12 show some example slices of the reconstructed absorption coefficients at certain depths, along with the ground truths with a higher resolution of 0.1 × 0.1 cm^2^, for patients 06L, 08L, 08R, 10R, and 14L respectively. In each figure, columns 1–4 correspond to the Tikhonov method, CS method with L1-norm, CS method with TV, and ground truth respectively. Visual comparisons between the reconstruction results by the three methods can be conducted from these figures. In Figs. 8–12 for patients 06L, 08L, 08R, 10R, and 14L, the slices of absorption coefficients reconstructed by all the three methods have good structural similarity with the ground truths. Optical images reconstructed by all three methods showed the vessel tubular structures very well in lateral directions. However, the spatial variations along the depth direction were much slower in all reconstructed images than that of the ground truths. When these basic reconstruction methods were applied to the complex vasculatures, the reconstructed images are contaminated by artifacts and have poor contrast compared to the ground truths. A lot of work is still demanded to improve the image reconstruction performance when it comes to complex structures. By comparing the results for patients 06L, 08L, 08R, 10R, and 14L by the three methods, one can see that the CS methods with L1-norm and TV performed better than the Tikhonov method in terms of contrast. In our future studies, the sparsifying transform could be used to further improve the performance of the CS methods. The performance difference between CS methods with L1-norm and TV is not significant, which suggests that these two regularization terms work equally well for the CS method in these cases.

## Data Availability

ImageJ2 was used for image crop, subtraction, and average. Hessian-based Frangi Vesselness filter^43^ was used for vessel enhancement. VMTK (http://www.vmtk.org) was used for vessel segmentation. ImageJ2 was used for breast and vessel 3D surface generation. ABAQUS/Standard 2018 was used for breast compression. Custom code (Matlab R2016b) ‘vesselDeform’ publicly available on *figshare*^27^ and stlTools^44^ were used for vessel deformation. SOLIDWORKS 2020 was used for breast and vessel surface combination. Autodesk Netfabb premium 2020 was used for deformed vessel surface mesh repair. COMSOL Multiphysics 5.5 was used for optical simulation. Custom code (Matlab R2016b) ‘imageReconstruction’ publicly available on *figshare*^27^ was used for image reconstruction.
